# Assessing ethical behavior and self-control in elite ultimate championships: a cross-sectional study using the spirit of the game scoring system

**DOI:** 10.3389/fspor.2024.1297821

**Published:** 2024-05-01

**Authors:** José Pedro Amoroso, Luís Coelho, Rebecca A. Boulton, Christie M. González-Toro, Felipe Costa, Efstathios Christodoulides, Wouter Cools, Dean Dudley, James E. Moore, Guilherme Eustáquio Furtado, Ming-Yang Cheng, Luís Calmeiro

**Affiliations:** ^1^ ESECS—Polytechnic of Leiria, Leiria, Portugal; ^2^ CIEQV—Life Quality Research Center, Polytechnic of Leiria, Leiria, Portugal; ^3^ Research Center in Sport, Health, and Human Development (CIDESD), Vila Real, Portugal; ^4^ Biological and Environmental Sciences, University of Stirling, Scotland, United Kingdom; ^5^ Department of Kinesiology, Manhattan College, Bronx, NY, United States; ^6^ Faculty of Physical Education, Brasília University, Brasília, Brasil; ^7^ School of Sciences, Sport and Exercise Sciences, University of Central Lancashire Cyprus, Larnaka, Cyprus; ^8^ BrusselMultidisciplinair Instituut Lerarenopleiding, Brussels Institute for Teacher Education & Movement and Nutrition for Health and Performance (MOVE) Research Group, Vrije Universiteit, Brussels, Belgium; ^9^ Artevelde University of Applied Sciences, Ghent, Belgium; ^10^ Macquarie School of Education, Macquarie University, Sydney, NSW, Australia; ^11^ Department of Bioengineering, Imperial College London, London, United Kingdom; ^12^ Polytechnic Institute of Coimbra, Applied Research Institute, Coimbra, Portugal; ^13^ Research Centre for Natural Resources Environment and Society (CERNAS), Polytechnic Institute of Coimbra, Coimbra, Portugal; ^14^ School of Psychology, Beijing Sport University, Beijing, China; ^15^ National Institute of Education, Nanyang Technological University, Singapore, Singapore; ^16^ Faculty of Medicine, Institute of Environmental Health, University of Lisbon, Lisbon, Portugal

**Keywords:** self-refereeing, self-regulation, physical activity, moral competence, sportsmanship, self-control, ethical behavior

## Abstract

**Introduction:**

Implementing a self-refereeing system presents a unique challenge in sports education, particularly in academic and training settings where officiated sports prevail. However, Ultimate Frisbee stands out by entrusting players with both athlete and referee roles, introducing distinctive ethical complexities. This manuscript is intended to evaluate ethical behavior and self-control within the Spirit of the Game (SOTG) scoring system in Elite Ultimate. To address these, Ultimate employs the (SOTG) scoring system, integral since the sport's inception in the late 1980s. SOTG aims to enhance and evaluate athletes’ ethical conduct. This study evaluates SOTG's effectiveness in elite-level Ultimate, analyzing variations across divisions and age groups in three high-level tournaments.

**Methods:**

Using a cross-sectional design, data were collected from five international Ultimate tournaments in 2022. Teams spanned diverse age groups (under 17 to over 50) and divisions (women's, mixed, open). Post-match, teams assessed opponents’ SOTG in five domains: Rules knowledge, fouls, fairness, attitude/self-control, and communication. Ratings used a 5-point Likert scale (“poor” to “excellent”). An overall SOTG score was calculated by aggregating domain scores.

**Results:**

Our study consistently revealed high SOTG scores, reflecting strong sportsmanship. “Positive attitude and self-control” consistently ranked highest, while “Knowledge and use of the rules” scored lowest. Divisional differences in SOTG were statistically insignificant. Notably, WMUCC2022 (participants aged 30+) had significantly higher SOTG scores, possibly indicating age-related self-control improvement or evolving sport culture. Lower rules knowledge scores may stem from linguistic translation challenges.

**Conclusion:**

Self-refereeing promotes ethical behavior across divisions and age groups. SOTG underscores sportsmanship's importance and aligns with International Olympic Committee (IOC) and with Sustainable Development Goals (SDGs), particularly SDG 3, 4, 5 and 16 fostering a fairer, healthier, and more peaceful world.

## 1 Introduction

This manuscript is intended ethical behavior and self-control within the Spirit of the Game (SOTG) Scoring System in Elite Ultimate Frisbee. The popular belief that sport builds character is almost as old as the origins of sport itself. The ultimate reflection of this philosophy, which has far-reaching implications for the development of morality, involves the concept of fair play. Fair play manifests itself in respect for the rules of the game, consideration for the opponent, honest competition, and the pursuit of enjoyment of the game itself (1, 2). Education is important in relation to a discussion regarding SOTG teaching in physical education and sports pedagogy, and important in relation to the question of who or what should be transformed in order for transformative learning and teaching to occur (3). In recent years, It has been argued that the educational value of sport has declined (4, 5). Violence and aggressive behaviour continue to be a part of sports culture that is relatively tolerated and to which does not seem to attach punishment (6). Sporting excellence should be gratifying for spectators as well as for athletes, not only because of outstanding athletic performance, but also due to their ethical qualities encompassing courage, self-control, generosity, and fairmindedness (7). These attributes have the potential to make sport more attractive from a spectator's perspective and increase its educational value for different target groups.

As the leader of the Olympic Movement, the IOC will continue to work to provide access to sport for people around the world. Over the past decade, many partnerships have been established with UN agencies to develop global campaigns, but also with local organisations through NOCs and NFs to increase sports participation at all levels of society. In 2015, the United Nations recognised sport as an important enabler for achieving the SDGs. This was welcomed by the Olympic Movement and the Sport for Development and Peace community with great interest and a commitment to further develop society through sport (8).

Self-regulation is the ability to purposefully regulate dominant impulses, needs and desires to allow individuals to attain desired long-term outcomes (9). Self-regulation is an important skill in sport participation and a multifaceted phenomenon operating through a number of subsidiary cognitive processes, including self-monitoring, standard setting, evaluative judgment, self-appraisal, and affective self-reaction (10). In most sports the presence of a referee or other official takes the forces the player to engage in self-control. An exception to this is Ultimate, non-contact team sport played with a flying disc. It is unique among team sports because it is self-refereed, even at the world championship level. The self-refereeing system used in Ultimate involves each team assessing their opponents and themselves after each match on various aspects of the “Spirit of the Game” (SOTG) such as Knowledge and use of the rules, Fouls and body contact, Fair-mindedness, Positive attitude and self-control, and Communication (11).

Good communication, sportspersonship, and respect are important reasons why people stay involved in sports (12). Within the physical activity and sport literature, communication has also been shown to contribute to team cohesiveness (13). Teams which demonstrate high SOTG behaviour will communicate better within the team, sharing knowledge and strategies effectively to achieve the best competitive outcomes (14). However, it has been argued that teams with poor SOTG are often seen as competitively superior, an assertion that remains to be formally tested. Communication is mandatory in Ultimate. It is through it that players communicate whenever a foul is called. Differentiating factor directly associated with the self-control that it is necessary to have/acquire in order to be able to expose our point of view.

Fairness is an important characteristic of ethical behaviour and moral reasoning. Beller and Stoll (15) argue that morality “involves a consideration of and concern for others, as well as being able to distinguish between what is honest and dishonest, fair, and unfair, respectful and disrespectful (p. 353). Haan (16) argued the importance of context in moral reasoning. Each sport is characterized by a particular complexity of relationships and roles that may have differing impact on the participants. Shields and Bredemeier (17) questioned athletes and non-athletes about moral dilemmas concerning everyday life and sport situations. Their research led to the development of the concept of bracketed morality. In sport situations, individuals condone behaviours that are not consistent with good character if demonstrated outside sport (18). For example, in “game reasoning”, players react with a lower moral reasoning stage during participation in sport, but, when they understand that issues are related to everyday life, they shift to more mature reasoning (19, 20).

Moreover, evidence exists that non-athletes present more mature moral reasoning than athletes (15) and that individual sport athletes, in turn, score higher than team sport athletes (21). Therefore, the contention that sport automatically builds character has been contested as competitive sport places athletes in conflict situations where sportsmanship and fair play is secondary to winning (15). In introducing SOTG system, Ultimate creates changes the nature of those complex relationships and is thought to provide a fairer, more honest sport experience.

In the current research, we seek to identify patterns of the athletes’ assessments of the SOTG a self-refereed sport such as Ultimate. Specifically, we compare SOTG scoring across levels of competition and divisions. In this study, the five international events encompass different age groups while the Divisions represent different gender composition, therefore acting as proxies for those variables. It has been argued that differences in moral reasoning exist depending on the stage of life (i.e., young adults, middle-aged adults) and gender, although longitudinal studies of moral dilemmas failed to identify differences between males and females. For example, contrarily to Gilligan's (1) conceptualisation, which holds that males have a normative and fairness orientation due to their focus on rights, duties and justice and females have a utilitarianism and perfectionism orientation due to their focus on welfare, relationships, caring and harmony, no gender differences were found in moral orientation (22) or stage (23). Therefore, the main research question we will address in this study is whether SOTG scores differ across competitions and divisions elite level Ultimate events, taking into consideration the overall SOTG scores and its different dimensions.

## 2 Methods

### 2.1 Study design

This study adopted a cross-sectional research design to evaluate the usefulness of the SOTG scoring system in elite-level Ultimate across various divisions and age groups. The study's cross-sectional approach allowed for the collection of data from multiple sources simultaneously, enabling an analysis of SOTG variations in diverse competitive settings.

### 2.2 Participants and settings

**Figure 1:**
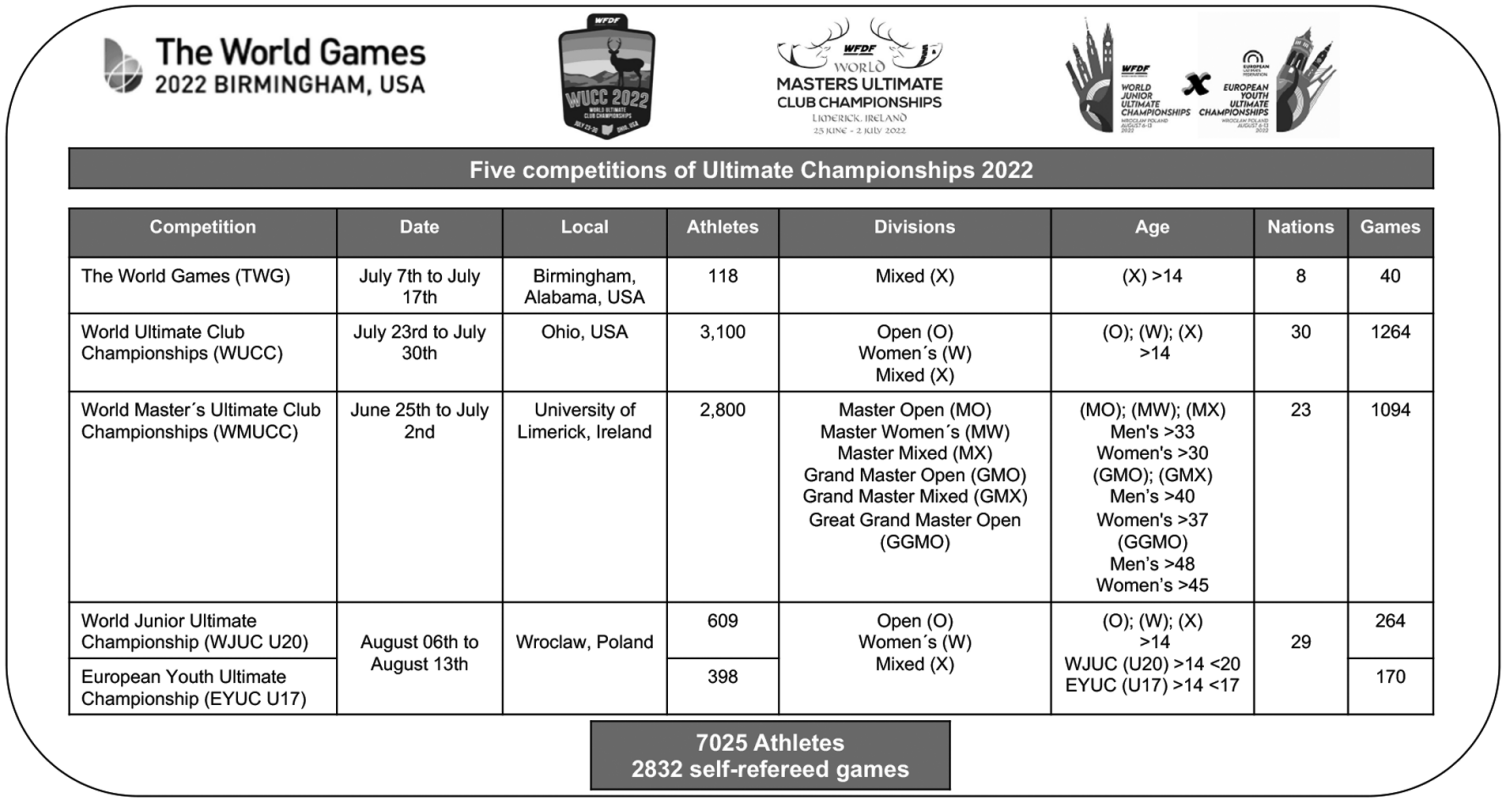
Overview participants and settings.

The participants in this study were drawn from a five competitions of Ultimate Frisbee tournaments, providing a rich dataset for our analysis. These tournaments included the World Games (TWG) held in Birmingham, Alabama, USA, from July 7th to July 17th, 2022, which brought together 118 athletes representing four continents: Asia (15), Europe (43), North America (30), and Oceania (14). The (24) World Ultimate Club Championships (WUCC), a major event in Ohio, USA, from July 23rd to July 30th, 2022, featured a substantial gathering of 3,100 athletes and 128 teams from 30 nations. Moreover, the (24) World Master's Ultimate Club Championships (WMUCC) convened at the University of Limerick, Ireland, from June 25th to July 2nd, 2022, attracting over 2,800 athletes and clubs hailing from 23 different nations. Lastly, the World and European Youth Championship (U20 & U17) jointly took place in Wroclaw, Poland, in 2022, where a total of 49 teams representing 29 national teams participated. All athletes are chosen by the national coaches of their teams. The culmination of these diverse tournaments resulted in a dataset comprising 2,832 self-refereed games, featuring 7,025 players (Figure 1). The players competed across nine divisions, each with specific age and gender criteria, including Open, Mixed, Women's, Master Open, Master Mixed, Master Women's, Grand Master Open, Grand Master Mixed, and Great Grand Master Open.

These divisions ensured a wide representation of participants across various age groups and genders, contributing to the richness of the dataset and enabling a comprehensive analysis of SOTG scores.

### 2.3 Data collection

Immediately after each game SOTG score sheets were collected by the Spirit Director of the event after: (a) the Spirit Captain (SC) facilitated a Spirit Circles (Sci) event with the opposing team. If for some reason there was no time to set up a Sci, the SCs at least checked in with the opposing team's SC to share any quick thoughts and to decide if further discussion was needed; (b) the teams evaluated their opponent teams promptly on the five principles of SOTG. The whole of each team was required to engage in scoring SOTG, to reflect on the game and their own team's spirit; (c) scores were entered or returned promptly to tournament organizers or scorekeepers; and (d) all SOTG scores were saved into a digital spreadsheet. During the tournaments, the spirit team, led by the spirit directors of the WUCC, WMUCC, TWG, U17 and U20 constantly monitored the scores and followed up with any teams that had displayed signs of poor spirit. All scores were locked and saved online where they could be viewed by all teams. The online versions make up the raw data analysed in the current study. Data from 2,832 self-refereed games were collected.

### 2.4 Instrumentation

The “Spirit of the Game” (SOTG) was used for the first time in 1980 and since then it has been part of all national and international championships, at a time when lifelong friendships created such strong personal ties that basic mutual respect was given. It is considered the number 1 rule of disc sports. SOTG was measured based on a scoring system in which athletes within a team score the opposition team after each game (12). SOTG was measured by the sum of the scores obtained in five questions addressing the following domains: Knowledge and use of the rules; Fouls and body contact; Fair-mindedness; Positive attitude and self-control, and Communication. Answers were given on a 5-point Likert scale (0 = Poor; 1 = Not Good; 2 = Good; 3 = Very Good; 4 = Excellent). After each game, players rated whether the other team was “better than,” “worse than,” or “the same as” a rival in a regular game, using the anchor “Good” as a baseline for comparison. The final SOTG score is the sum scoring/marking and may vary between 0 and 20, where a score of 10 is considered normal, good SOTG (24).

### 2.5 Ethical issues

Permission for data collection was sought from the World Flying Disc Federation and approved by the Chair of Ethics Committee of WFDF (8 February 2022). The procedures followed the Declaration of Helsinki and produced by the Association (25) for research with humans. In this study, just teams scores were analyzed, we did not use personal data, and only general anonymized data were analysed.

### 2.6 Analysis

Data analysis was conducted with the Statistical Package for Social Sciences (SPSS 28.0). No missing data was observed. SOTG Scores were summarized using descriptive statistics (mean and standard deviation). Our data, for all cases, was confirmed to be not normally distributed, after using Kolmogorov–Smirnov normality test. Differences on SOTG scores between championships and divisions were examined by performing a Kruskal-Wallis H test, followed by Bonferroni post-hoc test. The level of statistical significance was set at 0.05.

## 3 Results

Mean overall SOTG scores for each event and for all divisions were above 10 points (Table 1), which corresponds to an assessment of “good” or better (10.8 ± 1.9). Nonetheless, some 0's and 20's scores were given at WUCC2022 and WMUCC2022, respectively, but not representative for all scores and championships, in sum, self-referring maintains a good level of ethical conduct in high level ultimate. The master's competition WMUCC2022 had significantly higher overall SOTG scores than all others with 11.31 ± 1.8 points (*p* < 0.001), followed by WUCC2022 (10.47 ± 1.9 points), U20 (10.50 ± 1.9 points), U17 (10.31 ± 1.7 points) and TWG2022 (10.28 ± 1.3 points). WMUCC2022 also showed higher mean individual results across all SOTG dimensions, but statistically significant differences were observed for “Knowledge and use of the rules” (2.01 ± 0.5), “Fouls and body contact” (2.06 ± 0.6), and “Fair-mindedness” (2.43 ± 0.6; *p* < 0.001) except for TWG2022, for which differences were statistically non-significant (1.93 ± 0.3 points, 1.83 ± 0.4 points, 2.08 ± 0.5 points, respectively).

**Table 1:** SOTG scores, overall and for each dimension, for WUCC2022, WMUCC2022, TWG2022, U17 & U20.

		*N*	Mean	Std	Min.	Máx.	*P*
SOTG	WUCC2022	1,264	10.47	1.9	0	17	<0,001
	WMUCC2022	1,094	11.31	1.8	3	20	
	TWG2022	40	10.28	1.3	6	13	
	U17	170	10.31	1.7	3	14	
	U20	264	10.50	1.9	3	16	
	Total	2,832	10.78	1.9	0	20	–
Knowledge and use of the rules	WUCC2022	1,264	1.89	0.4	0	4	<0,001
	WMUCC2022	1,094	2.01	0.5	0	4	
	TWG2022	40	1.93	0.3	1	2	
	U17	170	1.79	0.5	0	3	
	U20	264	1.84	0.4	0	3	
	Total	2,832	1.93	0.5	0	4	–
Fouls and body contact	WUCC2022	1,264	1.92	0.5	0	4	<0,001
	WMUCC2022	1,094	2.06	0.6	0	4	
	TWG2022	40	1.83	0.4	1	2	
	U17	170	1.86	0.5	1	3	
	U20	264	1.9	0.6	0	3	
	Total	2,832	1.97	0.6	0	4	–
Fair-mindedness	WUCC2022	1,264	2.16	0.7	0	4	<0,001
	WMUCC2022	1,094	2.43	0.6	0	4	
	TWG2022	40	2.08	0.5	1	3	
	U17	170	2.14	0.6	0	3	
	U20	264	2.15	0.7	0	4	
	Total	2,832	2.26	0.7	0	4	–
Positive attitude and self-control	WUCC2022	1,264	2.3	0.6	0	4	<0,001
	WMUCC2022	1,094	2.49	0.6	0	4	
	TWG2022	40	2.23	0.6	1	3	
	U17	170	2.44	0.7	1	4	
	U20	264	2.36	0.6	1	4	
	Total	2,832	2.38	0.6	0	4	–
Communication	WUCC2022	1,264	2.2	0.6	0	4	<0,001
	WMUCC2022	1,094	2.33	0.6	1	4	
	TWG2022	40	2.23	0.6	1	3	
	U17	170	2.08	0.5	1	3	
	U20	264	2.25	0.6	0	4	
	Total	2,832	2.25	0.6	0	4	–

For “Positive attitude and self-control”, WMUCC2022 had, once again the highest scores (2.49 ± 0.6 points), but it was only statistically different between WUCC2022 (2.3 ± 0.6 points) and U20 (2.36 ± 0.6 points). “Communication” was also scored most highly at WMUCC2022 (2.33 ± 0.6 points), but it was only statistically different between WUCC2022 (2.2 ± 0.6 points) and U17 (2.08 ± 0.5 points) (*p* < 0.001).

When SOTG scores are analysed within tournaments and compared across divisions for WUCC2022 (Table 2), there were small but statistically significant differences in overall mean SOTG scores. The Open division had the highest score (10.79 ± 1.9 points) and this was significantly higher than scores for the Mixed division (10.43 ± 2.2 points; *p* < 0.05) and the Women's division (10.21 ± 1.3 points; *p* < 0.001).

**Table 2:** Overall and detailed SOTG scores for WUCC2022, according to divisions.

Championship WUCC2022		*N*	Mean	Std	Min.	Máx.	*P*
SOTG	Open	398	10.79	1.9	0	17	<0.001
	Women's	394	10.21	1.3	5	14	
	Mixed	472	10.43	2.2	0	16	
	Total	1,264	10.47	1.9	0	17	–
Knowledge and use of the rules	Open	398	1.94	0.4	0	4	0.15
	Women's	394	1.89	0.4	0	3	
	Mixed	472	1.86	0.5	0	4	
	Total	1,264	1.89	0.4	0	4	–
Fouls and body contact	Open	398	1.97	0.6	0	3	0.116
	Women's	394	1.89	0.4	0	4	
	Mixed	472	1.91	0.6	0	4	
	Total	1,264	1.92	0.5	0	4	–
Fair-mindedness	Open	398	2.22	0.7	0	4	<0.001
	Women's	394	2.05	0.6	0	3	
	Mixed	472	2.20	0.7	0	4	
	Total	1,264	2.16	0.7	0	4	–
Positive attitude and self-control	Open	398	2.32	0.7	0	4	0.164
	Women's	394	2.25	0.5	0	4	
	Mixed	472	2.32	0.7	0	4	
	Total	1,264	2.30	0.6	0	4	–
Communication	Open	398	2.33	0.6	0	4	<0.001
	Women's	394	2.13	0.5	0	3	
	Mixed	472	2.14	0.6	0	4	
	Total	1,264	2.20	0.6	0	4	–

In the “Knowledge and use of the rules”, “Fouls and body contact” and “Positive attitude and self-control” dimensions, all three divisions obtained statistically similar results (total: 1.89 ± 0.4 points, 1.92 ± 0.5 points, and 2.30 ± 0.6 points, respectively; *p* > 0.05). On the other hand, “Fair-mindedness” and “Communication” were scored higher in the Open division. The Open scores for both dimensions were significantly higher than those given in the women's division. For mixed vs. open only fair-mindedness differed significantly (see Table 2 for a full breakdown).

Similar results were observed for WMUCC2022, but these can be further broken down into six age-gender categories (Table 3). Open divisions scored consistently the highest and were overall highest in the “Great Grand Master Open” division (12.44 ± 2.5 points; *p* < 0.001) followed by “Grand Master Open” (11.53 ± 1.7 points), “Master Open” (11.41 ± 1.7 points), “Grand Master Mixed” (11.28 ± 1.8 points), “Master Mixed” (11.22 ± 1.7 points) and “Master Women's” (10.52 ± 1.4 points). It is worth noting the fact that “Master Women's” division lower mean results are also statistically different from all other divisions (*p* < 0.001).

**Table 3:** Overall and detailed SOTG scores for WMUCC2022, according to divisions.

		*N*	Mean	Std	Min.	Máx.	*P*
SOTG	Master open	264	11.41	1.7	8	17	<0.001
	Master women's	190	10.52	1.4	5	13	
	Master mixed	288	11.22	1.7	3	18	
	Grand master open	146	11.53	1.7	6	15	
	Grand master mixed	102	11.28	1.8	5	15	
	Great grand master open	104	12.44	2.5	7	20	
	Total	1,094	11.31	1.8	3	20	–
Knowledge and use of the rules	Master open	264	2.02	0.5	0	4	<0.001
	Master women's	190	1.89	0.4	1	3	
	Master mixed	288	1.95	0.5	0	3	
	Grand master open	146	2.06	0.4	1	3	
	Grand master mixed	102	1.99	0.5	1	3	
	Great grand master open	104	2.34	0.7	1	4	
	Total	1,094	2.01	0.5	0	4	–
Fouls and body contact	Master open	264	2.08	0.6	1	3	<0.001
	Master women's	190	1.94	0.5	0	3	
	Master mixed	288	2.02	0.6	0	4	
	Grand master open	146	2.16	0.6	1	3	
	Grand master mixed	102	2.01	0.6	0	3	
	Great grand master open	104	2.23	0.7	1	4	
	Total	1,094	2.06	0.6	0	4	–
Fair-mindedness	Master open	264	2.46	0.6	1	4	<0.001
	Master women's	190	2.18	0.6	1	4	
	Master mixed	288	2.42	0.7	0	4	
	Grand master open	146	2.45	0.6	1	4	
	Grand master mixed	102	2.51	0.7	1	4	
	Great grand master open	104	2.68	0.6	1	4	
	Total	1,094	2.43	0.6	0	4	–
Positive attitude and self-control	Master open	264	2.50	0.6	1	4	<0.001
	Master women's	190	2.33	0.6	1	4	
	Master mixed	288	2.58	0.6	0	4	
	Grand master open	146	2.45	0.6	0	3	
	Grand master mixed	102	2.46	0.6	0	3	
	Great grand master open	104	2.59	0.7	1	4	
	Total	1,094	2.49	0.6	0	4	–
Communication	Master open	264	2.36	0.6	1	4	<0.001
	Master women's	190	2.18	0.4	1	3	
	Master mixed	288	2.26	0.5	1	4	
	Grand master open	146	2.41	0.5	1	4	
	Grand master mixed	102	2.31	0.6	1	4	
	Great grand master open	104	2.61	0.7	1	4	
	Total	1,094	2.33	0.6	1	4	–

In the “Knowledge and use of the rules” dimension, “Great Grand Master Open” division had the highest score when compared with all the others, with a mean score of 2.34 ± 0.7 points (*p* < 0.001). “Master Women's” achieved, once again, the lowest mean score for this dimension (1.89 ± 0.4 points), nevertheless, it was only statistically different from “Grand Master Open” (*p* < 0.05) and “Great Grand Master Open” divisions (*p* < 0.001).

For “Fouls and body contact” “Great Grand Master Open” division again had the highest score when compared with all the others with a mean score of 2.23 ± 0.7 points (*p* < 0.001) and this was statistically different from “Master Women's” (1.84 ± 0.5 points, *p* < 0.001) and “Master Mixed” divisions (2.02 ± 0.6 points *p* < 0.05).

In the Fair-mindedness dimension, “Great Grand Master Open” division again had the highest score when compared with all the others, with a mean score of 2.68 ± 0.6 points, this results only showed to be statistically like “Grand Master Open” and “Grand Master Mixed” (*p* > 0.05). “Master Women's” gathered, once again, the lowest mean score for this dimension (2.18 ± 0.6 points) and it was statistically different from all others (*p* < 0.001).

“Positive attitude and self-control” and “Communication” dimensions had “Great Grand Master Open” division scores, once again, on top of all other divisions with mean results of 2.59 ± 0.7 and 2.61 ± 0.7 points, respectively. Nonetheless, for the first, statistical differences were only confirmed between “Master Women's” (2.33 ± 0.6 points, *p* < 0.001), while on the second one was confirmed between all divisions (*p* < 0.001).

“Master Women's” received the lowest mean score for these two dimensions (2.33 ± 0.6 and 2.18 ± 0.4 points, respectively) but it was only statistically different from “Master open” (2.50 ± 0.6 points, *p* < 0.05), “Master mixed” and “Great Grand Master Open” (*p* < 0.001) for “Positive attitude and self-control” and statistically different from “Master open” (2.36 ± 0.7 points, *p* < 0.01), “Grand master open” (2.41 ± 0.5 points, *p* < 0.01) and “Great Grand Master Open” (*p* < 0.001) for “Communication” dimension.

## 4 Discussion

The aim of the study was to compare athletes’ SOTG scoring across levels of competition and divisions in high level Ultimate. Our results suggest this system may help in the construction of an ethical and sportspersonlike conduct across all levels and divisions, as overall average scores were all above 10 (good) and individual domain specific scores above or close to 2 (good). Importantly, these results suggest that self-refereeing is possible across different levels of play, at elite level competitions.

The International Olympics Committee (IOC) implicitly values SOTG through its core values of peace and development through sport. Yet, there still appears to be an underlying ambiguity about the “spirit” of Ultimate that remains intangible (26). Also in these competitions, the SOTG is valued positively in all divisions (open, womeńs and mixed) at the Joint Junior Ultimate championship (11). A self-refereed sport like Ultimate has the potential to develop pedagogies which teach self-regulation, moral reasoning and communication whilst improving wellbeing through the physical benefits of activity. Therefore, Ultimate has the potential to promote teamwork, task cohesion, leadership, and increase friendship (27). As such, self-refereeing and SOTG scoring may be employed as a tool for developmental and social education of young people and further contribute to the use of sport experiences to develop athletes’ life skills (28, 29). Establishing local knowledge is essential before attempting to engage new participants in the sport, particularly working with disadvantaged young people (30, 31).

The results of our study also highlight some potentially interesting patterns within divisions. Our finding that SOTG scores were always above average (“good”) across all divisions, even when scores from under 17 and under 20 competitions were included, is encouraging and suggests that self-refereeing and SOTG scoring has potential as a teaching tool in sports education from a young age (U17). Although we found that all overall mean scores were “good” or better regardless of age group or gender split, we did observe scores to increase in older age categories, particularly for male dominated “Open” divisions. We also saw that scores were generally higher in Open divisions than Women's or Mixed (gender) divisions. We can only speculate as why this might be. One possibility is that gendered stereotypes about morally appropriate behaviour differ for male vs. female-identifying players. Women may be perceived as being less spirited than men when behaving the same.

This phenomenon has been highlighted in some studies in which assertive behaviour is evaluated more negatively when displayed by women compared to when identical behaviour is displayed by men (32, 33). During coeducational physical education, girls are more frequently confronted with a contradiction between further developing their female gender identity, and on the other hand the male gender role expectations such as competition, achievement orientation and self-reliance (34). It is also possible that women rate SOTG offences more harshly than men. It will be important in the future to carry out randomised control trials that ask participants of different gender identities to rate the SOTG of hypothetical players of different gender identities, in order to better understand how gender stereotypes influence the attribution of SOTG. Such studies could also evaluate the role of other biases, such as country or age, in SOTG scoring which will help when developing instructional materials to raise awareness about unconscious biases in SOTG scoring so that the process itself can be fairer.

Higher SOTG scores (and greater variability of scores) in the upper master's division suggests that a new generation of players, particularly those with more international competition experience, know that a good game is a 10 (normal game), and they must justify giving anything well outside that range (high or low). At The World Games data players had shown how to use the scoring system. Teams that lack experience with SOTG scoring often have to be told not to give high or low scores, unless there is some clear justification.

### 4.1 Strengths and limitations

The study's strengths lie in its comprehensive dataset from five international Ultimate tournaments, enabling a thorough examination of the SOTG scoring system's effectiveness. The cross-sectional design facilitated a diverse age and division analysis, highlighting the impact of self-refereeing and SOTG on ethical behavior in elite Ultimate. Noteworthy limitations include linguistic barriers affecting SOTG scores, necessitating caution in non-English-speaking regions. While gender biases in SOTG scoring were explored, other studies should delve into biases linked to factors like country and age. Complexity within the SOTG scoring system warrants further investigation.

### 4.2 Perspectives for future studies

Future research should focus on gender stereotypes’ influence on SOTG scoring and explore how varying behaviors are perceived based on gender. Raising awareness of unconscious biases in SOTG scoring and developing equitable assessment tools are essential. Comparative sports analysis, examining SOTG's applicability in other sports, is a promising avenue. Longitudinal studies tracking young athletes’ ethical development, emphasizing self-regulation and moral reasoning, are worth pursuing (35). Firstly, control trials of a more qualitative nature to better understand why scoring differences occur, especially checking for potential biases regarding gender, age, and nationality; sencondly, examining the applicability of SOTG and self-refereeing in other sports; and thirdly longitudinal studies tracking athletes’ development.

### 4.3 Practical implications

Integrating the SOTG system into sports education programs teaches crucial skills like self-regulation and ethical decision-making. This approach enhances young athletes’ well-being and fosters teamwork, leadership, and lasting friendships. On the other hand, the SOTG system's relevance extends to elite-level athletic development, maintaining ethical standards and promoting fair play. In international competitions, it serves as a universal ethical benchmark, ensuring ethical behavior in diverse cultural contexts.

Furthermore, recognizing SOTG's alignment emphasising its potential impact on broader societal objectives like peace, social inclusion, and sustainable development. Promoting ethical behavior in sports and sustainable development, aligns with COI and SDGs incorporating SOTG principles into sports education, fostering individuals who value fairness, cooperation, and respect, central to the SDG´s vision, particularly SDG 3 (Good Health and Well-being), SDG 4 (Quality Education) and SDG 5 (Gender Equality) and SDG 16 (Peace, Justice, and Sustainable Institutions) (11, 36). Ethical sports behavior contributes to peace, social inclusion, and sustainable development.

## 5 Conclusions

This study examined SOTG results in five different competitions. Score distributions aligned with expectations, assuming that most teams consistently exhibited good sportsmanship. “Positive attitude and self-control” consistently received the highest scores across all divisions, confirming positive SOTG outcomes in all competitions. In contrast, rules knowledge consistently scored the lowest in all divisions, emphasizing the need to enhance rule comprehension. Further data analysis may reveal the extent of linguistic barriers posing a problem in this regard. Utilizing the SOTG system through self-arbitration has proven to be an effective means of implementing self-refereeing and maintaining ethical conduct in a sporting context.

Furthermore, this study underscores the crucial role of sportsmanship and ethics through promoting ethical behaviour in sports and nurturing skills in self-regulation and moral decision-making, this study emphasizes sports’ contribution to creating a fairer, healthier, and more peaceful world.

## Data Availability

The original contributions presented in the study are included in the article/Supplementary Material, further inquiries can be directed to the corresponding author/s.
